# Cell Layers: uncovering clustering structure in unsupervised single-cell transcriptomic analysis

**DOI:** 10.1093/bioadv/vbac051

**Published:** 2022-08-04

**Authors:** Andrew P Blair, Robert K Hu, Elie N Farah, Neil C Chi, Katherine S Pollard, Pawel F Przytycki, Irfan S Kathiriya, Benoit G Bruneau

**Affiliations:** Biological and Medical Informatics Graduate Program, University of California, San Francisco, CA 94143, USA; Gladstone Institutes, San Francisco, CA 94158, USA; Division of Cardiology, Department of Medicine, University of California, San Diego, CA 92093, USA; Division of Cardiology, Department of Medicine, University of California, San Diego, CA 92093, USA; Biomedical Sciences Graduate Program, University of California, San Diego, CA 92093, USA; Division of Cardiology, Department of Medicine, University of California, San Diego, CA 92093, USA; Institute for Genomic Medicine, University of California, San Diego, CA 92093, USA; Gladstone Institutes, San Francisco, CA 94158, USA; Chan-Zuckerberg Biohub, San Francisco, CA 94143, USA; Bakar Computational Health Sciences Institute, University of California, San Francisco, CA 94143, USA; Institute for Human Genetics, University of California, San Francisco, CA 94143, USA; Department of Epidemiology and Biostatistics, University of California, San Francisco, CA 94143, USA; Quantitative Biology Institute, University of California, San Francisco, CA 94143, USA; Gladstone Institutes, San Francisco, CA 94158, USA; Gladstone Institutes, San Francisco, CA 94158, USA; Department of Anesthesia and Perioperative Care, University of California, San Francisco, CA 94143, USA; Gladstone Institutes, San Francisco, CA 94158, USA; Roddenberry Center for Stem Cell Biology and Medicine, Gladstone Institutes, San Francisco, CA 94158, USA; Cardiovascular Research Institute, University of California, San Francisco, CA 94143, USA; Department of Pediatrics, University of California, San Francisco, CA 94143, USA

## Abstract

**Motivation:**

Unsupervised clustering of single-cell transcriptomics is a powerful method for identifying cell populations. Static visualization techniques for single-cell clustering only display results for a single resolution parameter. Analysts will often evaluate more than one resolution parameter but then only report one.

**Results:**

We developed Cell Layers, an interactive Sankey tool for the quantitative investigation of gene expression, co-expression, biological processes and cluster integrity across clustering resolutions. Cell Layers enhances the interpretability of single-cell clustering by linking molecular data and cluster evaluation metrics, providing novel insight into cell populations.

**Availability and implementation:**

https://github.com/apblair/CellLayers.

## 1 Introduction

Single-cell RNA sequencing (scRNA-seq) technology allows for the classification of heterogeneous cell populations. Interpreting scRNA-seq profiles requires clustering to group cells with similar transcriptional signatures (Hwang *et al.*, 2018). Clustering is commonly implemented with graph-based modularity optimization methods. The number of clusters and subsequent cell-type characterization typically depends on an algorithm’s optimization method and benefit function. For example, modularity is a benefit function used to optimize the measurement of inter and intra-cluster for community detection in graph-based scRNA-seq clustering. Modularity is subject to a resolution limit, described as the minimum community size detected by optimizing modularity (Fortunato and Barthelemy, 2006). As a result, the resolution parameter regulates the number of clusters, with low values producing a few large clusters and higher values creating many small clusters (Traag *et al.*, 2019). A primary hurdle lies in providing a quantitative representation and explanation of cell-type relationships in multi-resolution clustering.

The Louvain algorithm is the basis for graph modularity optimization techniques. The method consists of a local moving and aggregation stage for finding a locally optimal solution for community detection (Blondel *et al.*, 2008). The Smart Local Moving (SLM) algorithm overcomes the Louvain local optimal solution limitation by allowing merged communities to split when beneficial (Waltman and van Eck, 2013). In scRNA-seq analysis, SLM normally operates on a shared nearest neighbor (SNN) graph that integrates similarity structure information (Zhu *et al.*, 2020). The most recent advancement to this approach is the Leiden algorithm, which solves the Louvain limitation of disconnected communities (Traag *et al.*, 2019). Future improvements to graph-based community detection that optimize modularity will benefit from data visualization techniques that evaluate the resolution parameter.

Alternative tools for multi-resolution cluster analysis, such as the R library clustree (Zappia and Oshlack, 2018), construct a non-interactive hierarchical tree representation of how communities split at different clustering resolutions. A single arrow and color opacity represent cell movement across clustering as a proportion ratio, which can be thresholded to remove edges. An alternative visualization is a Sankey diagram, where the width of arrows is proportional to a flow rate that indicates an increase or decrease in values. Sankey plots are helpful in the investigation of microbial communities. For example, the Google Charts tool called BioSankey (Platzer *et al.*, 2018) allows users to visualize the expression of single genes or species. In single-cell analysis, Sankey plots are used to depict cell-to-cell communication interactions, as illustrated by Cell Chat (Zhang *et al.*, 2021). However, none of these tools allow analysts to interact with the diagrams directly, and they have limited functionality in terms of how gene expression is overlaid.

We developed Cell Layers, an interactive data visualization tool for scRNA-seq multi-resolution cluster analysis. The output of Cell Layers is a Sankey network, and the network’s nodes are clusters. We call the edges between nodes the ‘flow’, which represents the transfer of cluster assignments of cells across a parameter grid search for clustering. Nodes can be colored by cluster evaluation metrics or biological activity. Similarly, edges can be colored by gene expression or co-expression. A ternary chart can also be used to interpret the co-expression of marker genes for each flow. The Sankey representation of multi-parameter clustering enables the rapid simultaneous evaluation of various resolutions for single-cell-type characterization.

A Sankey network is an intuitive visualization for multi-resolution clustering because the representation demonstrates the total volume for the flow of cells. In addition, Cell Layers advances multi-resolution clustering for single-cell analysis by providing analysts with a web portable and Jupyter-enabled interactive application, integration with scikit-learn’s cluster evaluation performance metrics, a method for tracing stable cell communities across cluster parameters, marker gene analysis of cell communities between clusters and co-expression of marker genes.

## 2 Methods

### 2.1 Clustering strategies

In a typical scRNA-seq analysis, clustering methods operate on a cell-to-cell *k*-nearest neighbor (kNN) graph. In Cell Layers, the default construction of a kNN graph is based on the Euclidean distance of a user-defined PCA subspace (Blondel *et al.*, 2008). For modularity and hierarchical clustering methods, cells are then iteratively grouped to optimize a modularity function thresholded by a resolution parameter. Cell communities are numerically labeled, with lower numbers signifying larger clusters. The output of a multi-resolution analysis is a cell-by-resolution-parameter matrix, where values are the cluster assignments of cells at each clustering resolution.

### 2.2 Data input

Cell Layers requires as input (i) a cell-by-resolution-parameter matrix and (ii) the corresponding cell-by-gene expression matrix. While our examples illustrate the cluster parameter as a resolution value for modularity-based clustering techniques, Cell Layers is agnostic to the user-selected clustering method that has been utilized to generate these inputs. To provide options for generating these objects, Cell Layers includes an R library (SetupCellLayers) that generates a cell-by-resolution-parameter matrix from a scRNA-seq kNN graph using the popular Seurat SLM and SNN clustering methods.

### 2.3 Data representation

In Cell Layers, each column of nodes represents a community structure C_*v*__,__*j*_, where *v* represents clusters {0,1,.*n*} at resolution parameter *j* ∈ ℝ. The Sankey network’s columns are ordered by a linearly increasing cluster parameter specified by a user-defined range and increment *q*. Each column’s community structure C_*v*__,__*j*_ is represented by nodes that are scaled by the cluster sample size and arranged to minimize edge overlap. The edges or ‘flow’ between clusters is computed as the number of cells from C_*v*__,__*j*_ assigned to C_*v*__,__*j*__+__*q*_. The data structure generated by Cell Layers is a directed acyclic graph.

### 2.4 Single-cell features

**Fig. 1.**
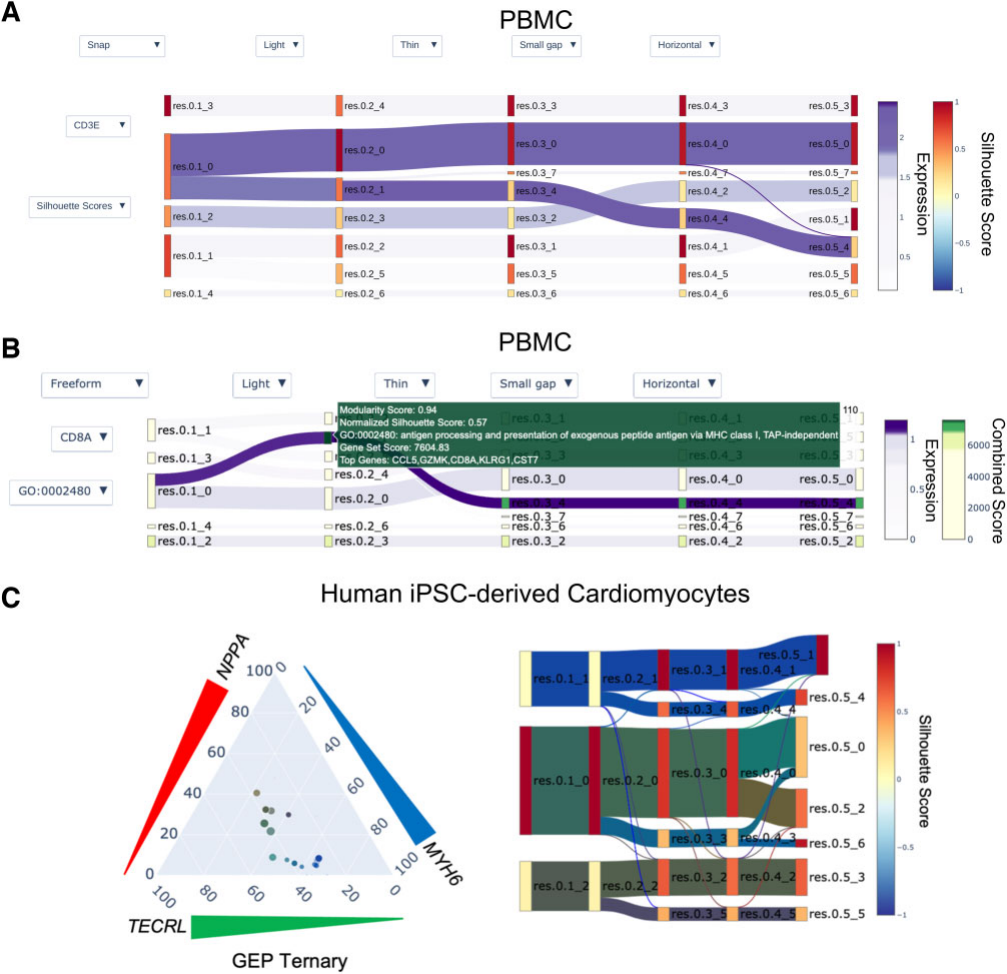
Application of Cell Layers on PBMCs or iPSC-derived cardiomyocyte differentiation. All nodes are labeled by their resolution parameter followed by an underscore indicating their cluster assignment. (**A**) PBMC multi-resolution analysis from 0.1 to 0.5. Edges are painted by CD3E, a marker gene for CD8 T, Memory CD4 T and Naive CD4 T cells. Nodes are painted by normalized Silhouette score. The lower Silhouette values indicate that samples are near the decision boundary of neighboring clusters. (**B**) Nodes painted by enrichR gene ontology (GO) 2018 Biological Process combined scores for GO: 0002480. The node-hover template provides users cluster performance metrics, GO term title, enrichR combined score and the top five differentially expressed genes. Edges are colored by the natural killer (NK) cell marker gene CD8A. (**C**) Multi-resolution analysis from 0.1 to 0.5 and marker gene co-expression of a dataset from iPSC-derived cardiomyocytes (Kathiriya *et al.*, 2021). The (left) ternary chart depicts the gene expression percentiles (GEPs) for the co-expression of *NPPA*, *MYH6* and *TECRL*. Each triangle on the outside of the ternary plot is oriented such that the base indicates increasing GEP. Each scatter point in the ternary chart specifies the co-expression of the genes (*NPPA*, *TECRL* and *MYH6*) as GEPs for a corresponding flow in the (right) Sankey plot. The size of a scatter point is a log2 transformation of the number of cells in its corresponding flow, then multiplied by a scalar value of two. The scatter point is generated by mapping each gene’s GEP. The Sankey nodes are painted by normalized Silhouette score, and edges are painted by the GEP of co-expressed *NPPA*, *TECRL* and *MYH6*

The flow may be painted by marker gene expression to facilitate cell-type characterization. Users can define several genes for their expression signature. To assess a single gene, each flow in the network is painted by the gene’s average expression for cells between C_*v*__,__*j*_ and C_*v*__,__*j*__+__*q*_ (Fig. 1A). Nodes may be painted by cluster metrics, gene ontology enrichment (Fig. 1B), or biological process activity (BPA). BPA uses the aREA method to transform gene expression levels using an ensemble of related genes to provide a robust representation of species-independent cellular states (Ding *et al.*, 2019).

Drop-down menus allow users to quickly switch between cluster evaluation metrics and BPA, which provide two orthogonal quantitative approaches for cell-type characterization. The first drop-down menu allows users to select a gene, dynamically updating the diagram’s flow and expression scale bar. The second drop-down menu allows the user to update node color by cluster metrics or BPA. Additional drop-down menus allow users to set node width and modify the network layout. Users may choose any Matplotlib colormap for painting the flow and nodes.

The co-expression of marker genes characterizes many cell types. To evaluate the co-expression for a user-defined geneset E, which contains *n*={3} genes over a flow with m cells, we created a gene expression percentage (GEP) for each gene E_*i*_. For a geneset E in a given flow, we calculate GEP as the ratio of the summed expression of a gene E_*i*__,__*j*_ over the total expression of all genes E_*k*__,__*j*_:

(1) $$GEP_{i}=\frac{\sum_{j=1}^{m}E_{i,j}}{\sum_{j=1}^{m}\sum_{k=1}^{n}E_{k,j}}.$$

The GEPs for a geneset are then mapped to a red, green or blue (RGB) hex code using Matplotlib. A Plotly ternary chart depicts the percentage ratios of co-expressed genes for each flow (Fig. 1C). Each point in the ternary plot corresponds to a flow’s sample size and expression percentage.

### 2.5 Future directions

Cell Layers was built for versatility, and its application extends beyond the single-cell features outlined here. Node-hover templates may include cluster metadata, which users may use to assess batch composition or integration. Downstream of multi-resolution analysis, Cell Layers can be used to evaluate data imputation models by assessing cluster stability metrics. Multi-resolution marker gene detection methods may be devised using statistical methods, such as the Jaccard Index. Additionally, protein activity profile methods could be integrated to resolve tissue-specific clusters (Ding *et al.*, 2018).

### 2.6 Software availability

Cell Layers is incorporated in the JupyterLab computing environment. Plotly is open-source software for data analytics and visualization of data science models. We made significant adaptations to Plotly’s interactive Sankey and Ternary API for scRNA-seq multi-resolution cluster analysis. Cell Layers will be available via pip, GitHub, Docker and Singularity.

## 3 Conclusion

Clustering of scRNA-seq data is used to identify cell populations computationally. Analysts typically assess multiple cluster parameters, cluster performance metrics and marker genes before annotating clusters. Fixed dimensionality reduction methods for visualizing single-cell clustering are limited by the number of attributes that users can assess. Cell Layers enables analysts to efficiently utilize multi-resolution parameters to explore and characterize single-cell populations interactively.

## Data Availability

The data underlying this article are available in GitHub at https://github.com/apblair/CellLayers and figshare at https://figshare.com/account/projects/136175/articles/19834771.
